# The age pattern of the male-to-female ratio in mortality from COVID-19 mirrors that of cardiovascular disease in the general population

**DOI:** 10.18632/aging.202639

**Published:** 2021-02-07

**Authors:** Ila Nimgaonkar, Linda Valeri, Ezra Susser, Sabiha Hussain, Jag Sunderram, Abraham Aviv

**Affiliations:** 1 Robert Wood Johnson Medical School, Rutgers State University of New Jersey, New Brunswick, NJ 08901, USA; 2 Department of Biostatistics, Mailman School of Public Health, Columbia University, New York, NY 10032, USA; 3 Mailman School of Public Health, Columbia University, New York, NY 10032, USA; 4 New York State Psychiatric Institute, New York, NY 10032, USA; 5 Department of Medicine, Robert Wood Johnson Medical School, Rutgers State University of New Jersey, New Brunswick, NJ 08901, USA; 6 Center of Human Development and Aging, New Jersey Medical School, Rutgers State University of New Jersey, Newark, NJ 07103, USA

**Keywords:** COVID-19, mortality, age, sex, cardiovascular disease

## Abstract

Males are at a higher risk of dying from COVID-19 than females. Older age and cardiovascular disease are also associated with COVID-19 mortality. To better understand how age and sex interact _in_ contributing to COVID-19 mortality, we stratified the male-to-female (sex) ratios in mortality by age group. We then compared the age-stratified sex ratios with those of cardiovascular mortality and cancer mortality in the general population. Data were obtained from official government sources in the US and five European countries: Italy, Spain, France, Germany, and the Netherlands. The sex ratio of deaths from COVID-19 exceeded one throughout adult life, increasing up to a peak in midlife, and declining markedly in later life. This pattern was also observed for the sex ratio of deaths from cardiovascular disease, but not cancer, in the general populations of the US and European countries. Therefore, the sex ratios of deaths from COVID-19 and from cardiovascular disease share similar patterns across the adult life course. The underlying mechanisms are poorly understood and warrant further investigation.

## INTRODUCTION

More males than females die from COVID-19, the disease caused by SARS-CoV-2. This was first observed in China, where 64% of COVID-19 deaths occurred in males [1]. As the epidemic spread worldwide, other countries similarly observed a higher percentage of deaths from COVID-19 in males [1]. The higher number of deaths among males is consistent with mortality patterns observed in several major viral epidemics/pandemics of the 20^th^ and 21^st^ centuries, including the Western African Ebola virus epidemic (2013-2016) [2] and the H1N1 Spanish Flu pandemic of 1918 [3]. Adult men also have an overall higher mortality rate than adult women from seasonal influenza based on data in the US between 1997-2007, with some variation depending on age group and underlying conditions [4].

In contrast, COVID-19 mortality by age differs from other viral pandemics. More than 80% of COVID-19 deaths in the US and European countries have occurred in adults older than 65 years [5], with very few deaths in young children [6]. Seasonal influenza causes relatively more pediatric deaths, especially in infants under the age of six months [7], in addition to a disproportionate number of deaths in older adults over the age of 65 years [8]. Several major viral pandemics of the 20^th^ and 21^st^ centuries have also shown different age-based mortality patterns from COVID-19. For instance, in the Spanish Flu of 1918 a large proportion of deaths were in young adults [9], and in the 2009 H1N1 influenza pandemic a large proportion occurred in children and adults under 60 years [10]. Therefore, COVID-19 mortality trends are consistent with sex-based, but not with age-based patterns seen in many other viral pandemics.

In searching for explanations for the distinctive pattern of deaths in COVID-19, we first examined variation by age group in the male-to-female (sex) ratio of mortality in data from the US and five European countries. Given that cardiovascular disease (CVD) has been strongly associated with increased risk of mortality in COVID-19 [11], we next examined variation in the sex ratio of CVD mortality by age group in the same countries. As CVD and cancer are the two major age-related disease categories that largely determine survival of adults in middle-and-high income societies [12], we further examined variation by age in the sex ratio of cancer mortality. Here we report our findings and offer potential explanations regarding their meaning.

## RESULTS

**Figure 1:**
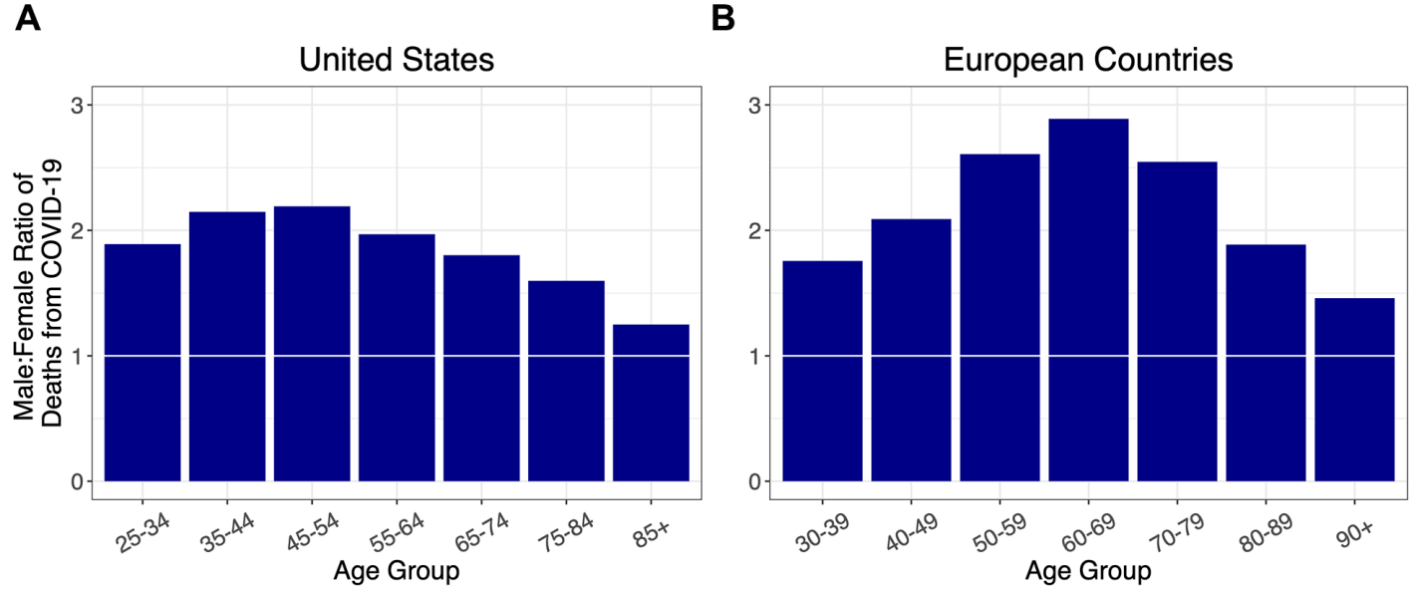
Ratios of male to female deaths from COVID-19 (adjusted for population sex distribution) for (**A**) the US, and (**B**) combined ratios for five European countries: Italy, France, Spain, Germany, and the Netherlands. A 1:1 ratio is indicated by white markers.

Stratified by age, and adjusted for population at risk in each age group (Supplementary Figure 1, Supplementary Table 2), the sex ratios for COVID-19 deaths among adults in the US and five European countries (Italy, Spain, France, Germany, and the Netherlands) showed similar overall patterns, initially rising to a peak in midlife and then falling (Figure 1). Specifically, the sex ratio of mortality peaked between the ages of 35-44 years in the US and 60-69 years in the European countries, and then progressively declined at older ages without dropping below one.

**Figure 2:**
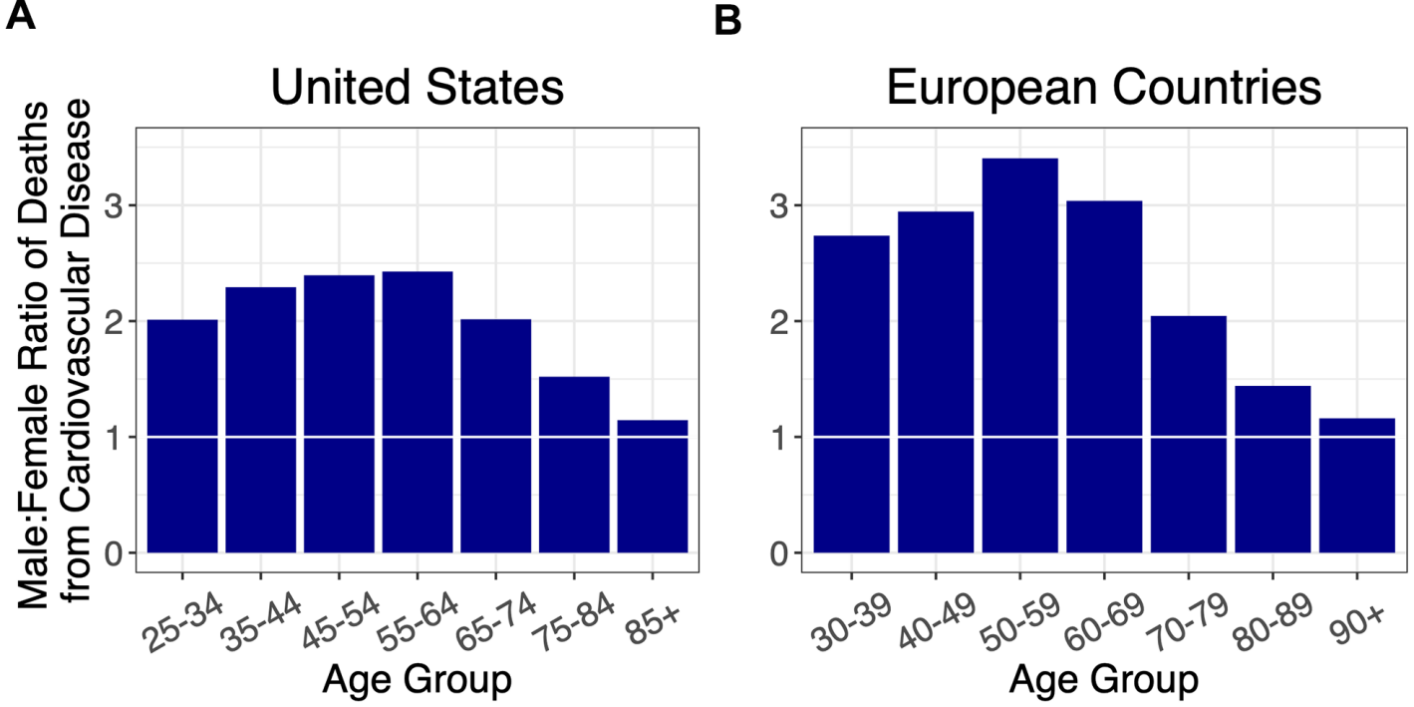
Ratios of male to female deaths from cardiovascular disease (adjusted for population sex distribution) for (**A**) the US, and (**B**) combined ratios for the European countries: Italy, France, Spain, Germany, and the Netherlands. A 1:1 ratio is indicated by white horizontal lines.

We next examined the sex ratio of CVD mortality by age in these countries (Figure 2, Supplementary Figure 2). The sex ratio for CVD mortality initially rose to a peak in midlife and then declined, in both the US and Europe. For ages younger than 70 years, the sex ratio for CVD mortality in Europe was higher than in the US.

**Figure 3:**
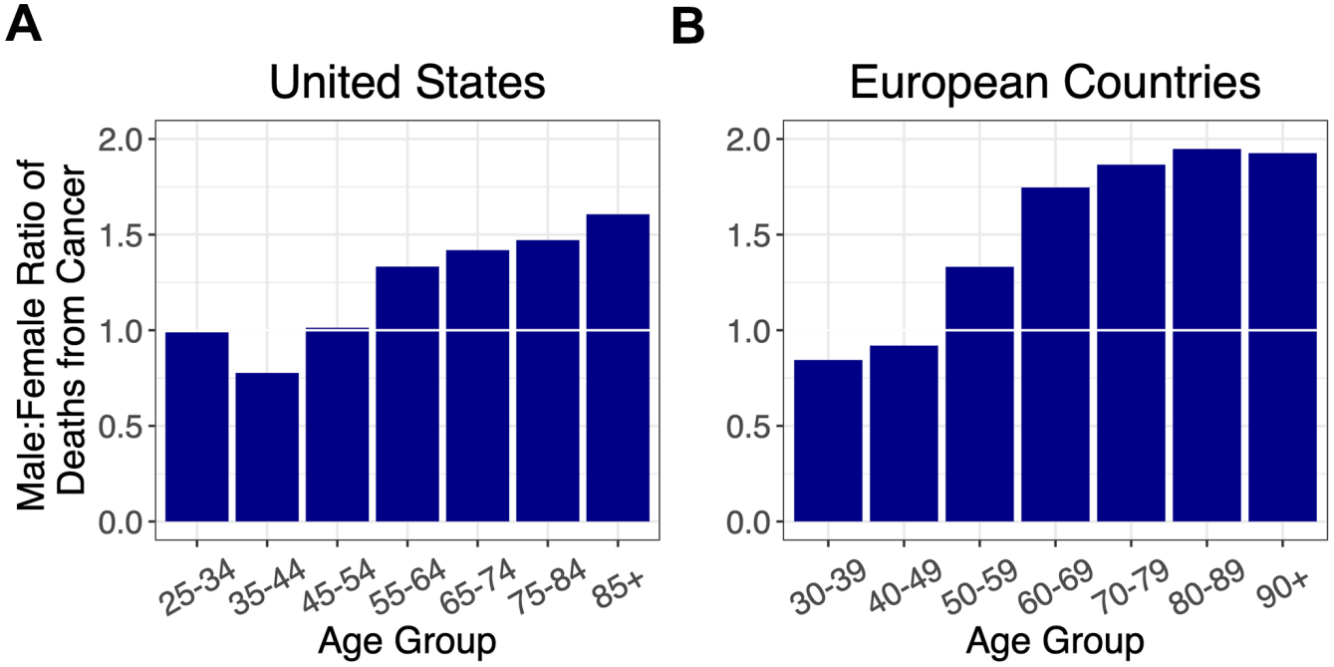
Ratios of male to female deaths from cancer (adjusted for population sex distribution) for (**A**) the US, and (**B**) combined ratios for the European countries: Italy, France, Spain, Germany, and the Netherlands. A 1:1 ratio is indicated by white horizontal lines.

As cancer is the other leading cause of adult mortality in the US and Europe [13], we also examined the sex ratio for cancer mortality by age (Figure 3, Supplementary Figures 3, 4). The sex ratio for cancer mortality increased after midlife with no evidence of decline thereafter.

**Figure 4:**
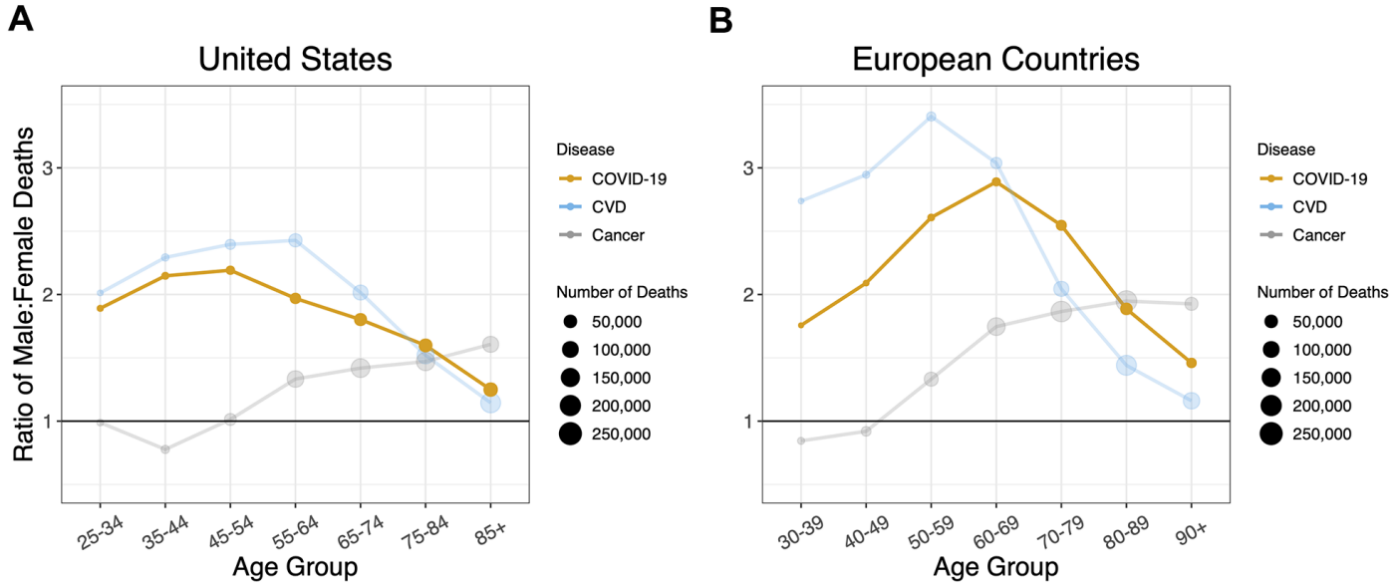
Sex ratios by age group in deaths for COVID-19, cardiovascular disease, and cancer in (**A**) the US and (**B**) the European Countries. A 1:1 ratio is indicated by black horizontal lines. Ratios are adjusted for sex distribution in the population at different age groups.

When the sex ratios of mortality by age for COVID-19, CVD and cancer were overlaid, the COVID-19 profile mirrored that of CVD, although, the sex ratio for CVD was much higher in Europe than the US for ages younger than 70 years (Figure 4). The profile of the sex ratio by age for cancer mortality, however, clearly differed from the ratios for COVID-19 and for CVD.

## DISCUSSION

### General considerations

Our key findings are as follows: (i) for all age groups, the death rate from COVID-19 was higher in males than females; (ii) the sex mortality ratio for COVID-19 rose to a peak in midlife and then narrowed with increasing age; and (iii) the sex mortality ratio for COVID-19 by age mirrored that for CVD but not cancer. Since the COVID-19 data were not linked to population databases with individual-level health information, we could not specifically examine the potential contribution of CVD to the age pattern of the sex mortality ratio from COVID-19. We propose, nonetheless, based on data displayed in Figure 4, that the sex ratio for COVID-19 mortality, particularly for adults older than 60 years, might partially reflect the mechanisms that drive the age pattern of the sex mortality ratio for CVD in the general population.

What then might be these mechanisms? The underlying biological reasons are likely multifactorial and complex. We review several potential explanations: pre-existing cardiovascular risk factors, sex hormones, and X chromosome mosaicism.

### Cardiovascular risk factors

Early available clinical data suggest that patients who become critically ill from COVID-19 are more likely to have pre-existing CVD risk factors such as hypertension and diabetes than patients who experience a milder disease course [14, 15]. Certain CVD risk factors also show different prevalence rates among males and females. For example, hypertension rates are higher in males than in females under the age of 60, but are not significantly different at ages 60 and over [16]. Males also have higher rates of both diagnosed and undiagnosed cases of diabetes, and typically develop atherosclerotic disease earlier than females [17]. These patterns for hypertension and atherosclerotic disease, which occur at higher rates in younger males and at more equal rates at older ages, share similarities with the sex ratios observed for COVID-19 and CVD mortality, and may partially explain the age pattern of the sex ratio in COVID-19 mortality. Though suggestive by our findings, definitive studies are needed to establish direct links between CVD risk factors and COVID-19 mortality.

### Sex hormones: estrogens and androgens

Endogenous estrogens have been associated with a protective effect against CVD in premenopausal women [18], while predisposing women to certain cancers [19]. Higher estrogen [20] and progesterone [21] levels in premenopausal women might also modulate immune responses and attenuate the severity of COVID-19. In this regard, data from the US show a decline in the sex ratio of COVID-19 mortality after ages 45-54 years, which coincides with the average age that women undergo menopause [22]. However, the data from the European countries show a drop in the sex ratio of COVID-19 mortality starting in the 60-69-year age group- later than the average age of menopause. Clinical trials are currently investigating whether estrogen or progesterone treatment can alleviate COVID-19 symptoms, which may provide more clarity to the role of ovarian hormones in COVID-19 pathogenesis (https://www.clinicaltrials.gov/ identifiers NCT04359329, NCT04365127).

Androgen increases the expression of *TMPRSS2* [23], a gene encoding Type II Transmembrane Serine Protease (TMPRSS2) which activates the SARS-CoV-2 spike protein, facilitating SARS-CoV-2 entry into cells [24]. A recent study suggests that androgen-deprivation therapies lowers the risk of SARS-CoV-2 infection [25]. Since testosterone level decreases with age, a higher level of the hormone in young and middle age men may upregulate *TMPRSS2* and thus contribute to the higher sex ratio for COVID-19 mortality before the sixth decade. That said, the knowledge of the effect of testosterone replacement therapy on the cardiovascular system is incomplete [26].

### X chromosome mosaicism

The two X chromosomes provide an advantage related to X-linked recessive diseases and other potentially deleterious mutations on the X chromosome. Random inactivation *in utero* of one X chromosome in each somatic cell engenders mosaicism that provides females with somatic cell diversity and the potential for selection of cells with an X chromosome harboring advantageous variant genes [27]. X chromosome mosaicism might be particularly advantageous for surviving infectious disease, since the X chromosome harbors a number of genes engaged in immune function [28] – therefore having two copies of these genes confers additional immunological diversity in women [29]. In addition, *ACE2*, the gene encoding angiotensin-converting enzyme 2, the cellular receptor for SARS-CoV-2 is on the X chromosome [30]. *ACE2* variants might play a role in left ventricular hypertrophy that is often the outcome of hypertension [31], which tracks with age [32]. Some of these variants might also influence CVD [33], but no association of *ACE2* polymorphisms with COVID-19 was observed thus far [34, 35].

### Limitations

Limitations to this study include: (a) potential differences in reporting COVID-19 deaths between the countries analyzed; (b) the lack of linked individual-level health and social databases; and (c) no information on the sex ratio of survival rates among people who acquired COVID-19. The latter would require, at a minimum, COVID-19 infection rates by age and sex in the general population (including asymptomatic infections) in order to determine whether there are sex- and age-based differences in survival from it.

Additionally, the study does not account for nuanced differences in the overall similar patterns for COVID-19 and CVD in the United States versus Europe. The peak sex ratio for COVID-19 occurs at an earlier age in the United States than Europe, and the sex ratio for CVD mortality is higher for Europe than the United States until the older age groups. Finally, we could not rule out that the lower sex ratios in COVID-19 and CVD for the oldest age groups were partly due to selection, in that males who survive to an exceptionally old age are “escapers” who hardly represent the general population of older males; in contrast, females who survive to such an old age might be “delayers” who largely represent the general population of older females [36, 37].

## CONCLUSIONS

Our analyses show similar trends in the sex ratio by age of mortality from COVID-19 and from CVD. We propose that these findings might be due to some shared underlying biological mechanisms. Individual-level data are essential to examine potential shared mechanisms and to establish the potential contribution of CVD to the age patterning of the sex ratio of mortality in COVID-19. We suggest that this line of research should be pursued and might uncover some of the causal mechanisms underlying COVID-19 mortality.

## MATERIALS AND METHODS

We focused our analysis on the US, Italy, Spain, France, Germany, and the Netherlands because of (a) availability of their sex- and age-disaggregated mortality data from COVID-19, (b) data stratification into age group bins of 10 years or less, and (c) a high number of cumulative deaths from COVID-19 (sources are shown in Supplementary Table 1). COVID-19 mortality data were retrieved from national databases including the US Centers for Disease Control, the Italian National Institute of Health, the French Institute for Demographic Studies, the Spanish Ministry of Health, the German Federal Ministry of Health, and the Dutch Ministry for Health, Welfare and Sport. The data were retrieved on October 14^th^, 2020 and reflect the cumulative COVID-19 deaths in each country from the beginning of the pandemic up to dates between May 22^nd^ – October 13^th^ 2020 (see Supplementary Tables 1, 2 for details). (Note that after May 22^nd^, the Spanish Ministry of Health stopped providing sex- and age-disaggregated data on COVID-19 deaths [38].) In the US, data were included for age groups 25 years and older, and stratified into 10-year bins. For the European Countries, data were included for age groups 30 years and above and stratified into 10-year bins, and deaths were combined across the five countries by age group and sex. Age groups below 25 years were excluded from the analysis due to the smaller number of cases. The population at risk in each age group was retrieved from populationpyramid.net, a website that aggregates data from the United Nations Department of Economic and Social Affairs, Population Division.

For the analysis of mortality from CVD and cancer, data were extracted from the World Health Organization Mortality Database (https://www.who.int/healthinfo/mortality_data/en/), which collects national data on deaths from civil registries. The Database contains number of deaths by country, year, sex, age group and cause of death, and population size by country, year, sex and age group. The causes of death are categorized by International Classification of Disease (ICD)-10 codes. CVD deaths principally included deaths from coronary heart disease (ICD-10 codes I20-I25) or stroke (I60-I69) [39]. Cancer death data included deaths from all neoplasms (C00-C97, D00-D48) (Supplementary Table 3). The non-sex-biased cancers (Supplementary Figure 4) included those indicated by ICD-10 codes C00-C49, C64-D04, D09-D23, D30-D38, and D41-D48. Data was extracted for France (2014), Germany (2015), Italy (2015), Netherlands (2016), Spain (2015), and the US (2015), with the year of the most recent data available indicated in parentheses (Supplementary Tables 4–6). Population adjustment was done using population data from the same years as the mortality data. These population data were also extracted from the World Health Organization Mortality Database.

Plots and data visualizations were created using the ggplot2 package in R (https://ggplot2.tidyverse.org).

## Supplementary Material

Supplementary Figures

Supplementary Tables 1 and 2

Supplementary Table 3

Supplementary Tables 4, 5 and 6
