# Supplementary material for: The age pattern of the male-to-female ratio in mortality from COVID-19 mirrors that of cardiovascular disease in the general population
Source: Aging (Albany NY). 2021 Feb 7;13(3):3190–201. doi: 10.18632/aging.202639 (PMC7906174; doi:10.18632/aging.202639)
Supplement: Supplementary Figures [file aging-13-202639-s001.pdf]

SUPPLEMENTARY FIGURES

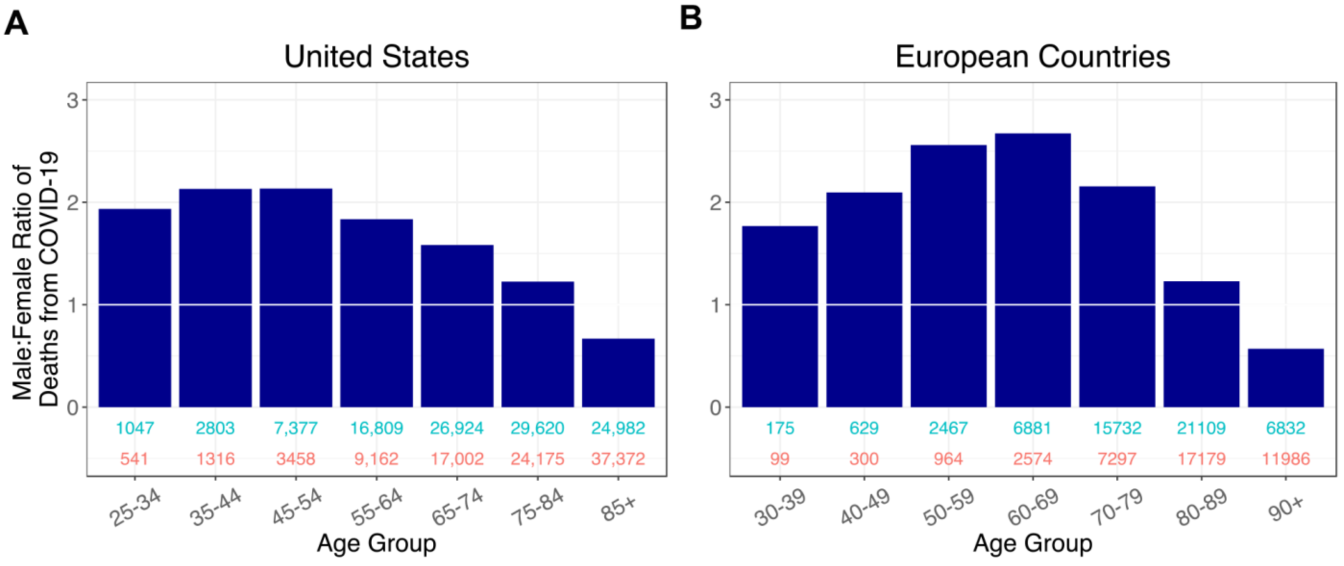

Supplementary Figure 1. Ratios of male to female deaths from COVID-19 (raw data) for the (**A**) United States, and (**B**) combined ratios for five European countries: Italy, France, Spain, Germany, and the Netherlands. A 1:1 ratio is indicated by white markers. Numbers below plots indicate number of male deaths (turquoise) and female deaths (orange).

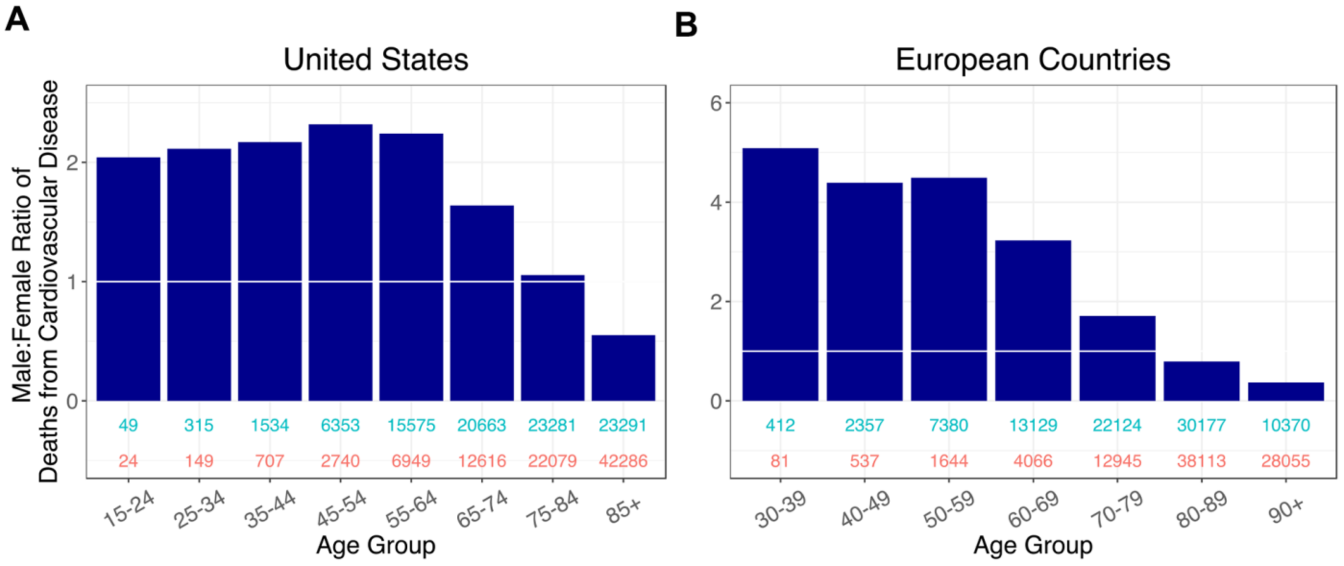

Supplementary Figure 2. Ratios of male to female deaths from cardiovascular disease (raw data) for the (**A**) United States, and (**B**) combined ratios for five European countries: Italy, France, Spain, Germany, and the Netherlands. A 1:1 ratio is indicated by white markers. Numbers below plots indicate number of male deaths (turquoise) and female deaths (orange). Note that y-axes have different scales.

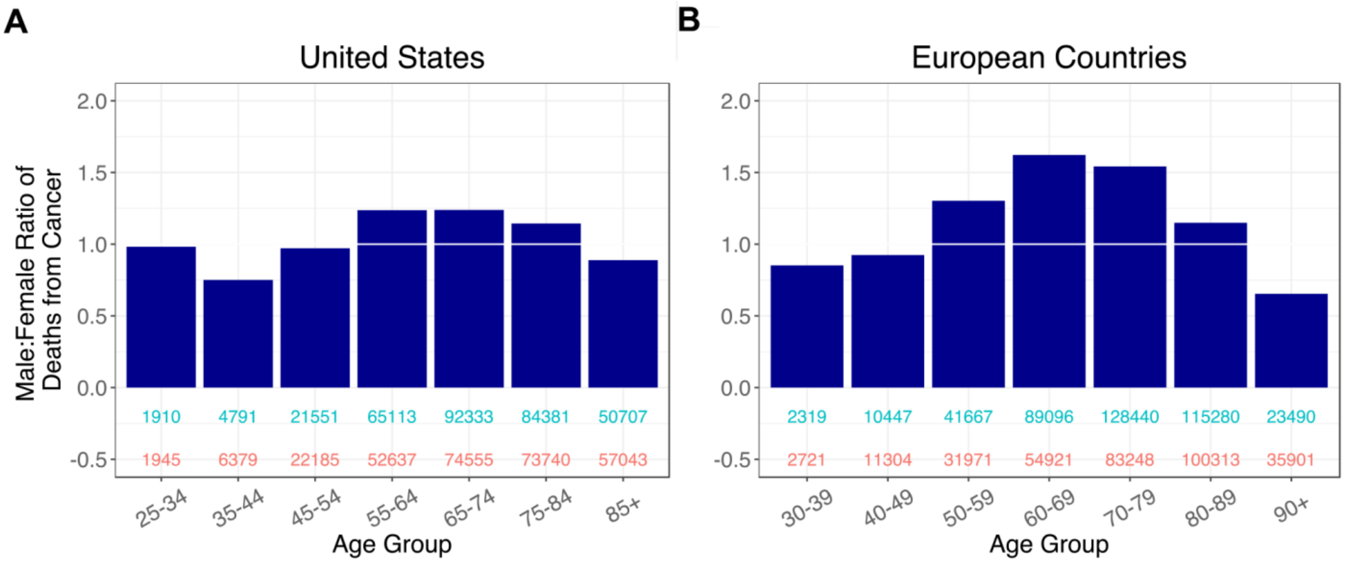

Supplementary Figure 3. Ratios of male to female deaths from cancer (raw data) for the (**A**) United States, and (**B**) combined ratios for five European countries: Italy, France, Spain, Germany, and the Netherlands. A 1:1 ratio is indicated by white markers. Numbers below plots indicate number of male deaths (turquoise) and female deaths (orange).

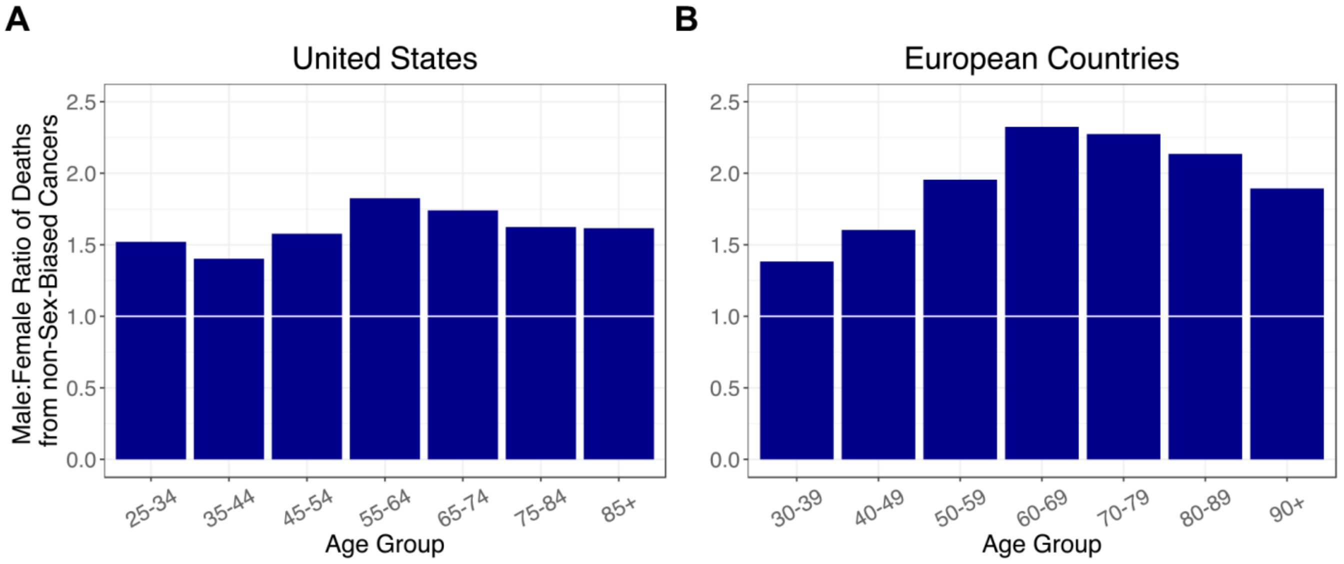

Supplementary Figure 4. Ratios of male to female deaths from non-sex-biased cancers (adjusted for population sex distribution) for the (**A**) United States, and (**B**) combined ratios for five European countries: Italy, France, Spain, Germany, and the Netherlands. A 1:1 ratio is indicated by white markers.
