# Supplementary material for: The age pattern of the male-to-female ratio in mortality from COVID-19 mirrors that of cardiovascular disease in the general population
Source: Aging (Albany NY). 2021 Feb 7;13(3):3190–201. doi: 10.18632/aging.202639 (PMC7906174; doi:10.18632/aging.202639)
Supplement: Supplementary Tables 1 and 2 [file aging-13-202639-s002.pdf]

SUPPLEMENTARY TABLES

Supplementary Table 1. Sources for COVID-19 deaths data.

Country	Website	Date updated	Date accessed
United States	https://data.cdc.gov/	Oct 10 2020	Oct 14 2020
Italy	https://www.epicentro.iss.it/	Oct 4 2020	Oct 14 2020
France	https://dc-covid.site.ined.fr/	Oct 8 2020	Oct 14 2020
Spain	https://www.mscbs.gob.es/	May 22 2020	Oct 14 2020
Germany	https://www.rki.de/	Oct 13 2020	Oct 14 2020
Netherlands	https://www.rivm.nl/	Oct 13 2020	Oct 14 2020

Supplementary Table 2. Raw data for COVID-19 deaths stratified by age and sex.

	Age group	Male	Female		Age group	Males	Females		Age group	Male	Female
**United States**	0-4	22	15	**Italy**	0-9	1	3	**France**	0-9	2	1
	5-14	24	13		10-19	0	0		10-19	2	2
	15-24	230	144		20-29	12	3		20-29	18	9
	25-34	1047	541		30-39	46	24		30-39	61	42
	35-44	2803	1316		40-49	229	89		40-49	178	94
	45-54	7377	3458		50-59	959	302		50-59	667	324
	55-64	16809	9162		60-69	2742	864		60-69	1803	751
	65-74	26924	17002		70-79	6505	2892		70-79	3321	1506
	75-84	29620	24175		80-89	8047	6677		80-89	4600	3415
	85+	24982	37372		90+	2101	4511		90+	1989	2741
	**Age group**	**Male**	**Female**		**Age group**	**Males**	**Females**		**Age group**	**Male**	**Female**
**Spain**	0-9	1	1	**Germany**	0-9	0	1	**Netherlands**	0-9	0	0
	10-19	3	2		10-19	1	0		10-19	1	0
	20-29	15	9		20-29	8	3		20-29	3	0
	30-39	42	21		30-39	18	8		30-39	8	4
	40-49	140	77		40-49	60	24		40-49	22	16
	50-59	467	192		50-59	265	96		50-59	109	50
	60-69	1283	539		60-69	691	244		60-69	362	176
	70-79	3326	1564		70-79	1467	708		70-79	1113	627
	80-89	4655	3792		80-89	2226	2006		80-89	1581	1289
	90+	1681	2723		90+	617	1185		90+	444	826
