# Supplementary material for: The age pattern of the male-to-female ratio in mortality from COVID-19 mirrors that of cardiovascular disease in the general population
Source: Aging (Albany NY). 2021 Feb 7;13(3):3190–201. doi: 10.18632/aging.202639 (PMC7906174; doi:10.18632/aging.202639)
Supplement: Supplementary Table 3 [file aging-13-202639-s003.doc]

**Supplementary Table** 3. International Classification of Diseases (ICD)-10 Codes for Causes of Death. Causes of death marked with an ‘X’ in column 2 were not included in the analysis of non-sex-biased cancers.

| **Cardiovascular Disease** | **Sex-biased** |
| --- | --- |
| I20 Angina pectoris |  |
| I21 Acute myocardial infarction |  |
| I22 Subsequent myocardial infarction |  |
| I23 Certain current complications following acute myocardial infarction |  |
| I24 Other acute ischaemic heart diseases |  |
| I25 Chronic ischaemic heart disease |  |
| I60 Subarachnoid haemorrhage |  |
| I61 Intracerebral haemorrhage |  |
| I62 Other nontraumatic intracranial haemorrhage |  |
| I63 Cerebral infarction |  |
| I64 Stroke, not specified as haemorrhage or infarction |  |
| I67 Other cerebrovascular diseases |  |
| I69 Sequelae of cerebrovascular disease |  |
|  |  |
| **Cancer** |  |
| C00 Malignant neoplasm of lip |  |
| C01 Malignant neoplasm of base of tongue |  |
| C02 Malignant neoplasm of other and unspecified parts of tongue |  |
| C03 Malignant neoplasm of gum |  |
| C04 Malignant neoplasm of floor of mouth |  |
| C05 Malignant neoplasm of palate |  |
| C06 Malignant neoplasm of other and unspecified parts of mouth |  |
| C07 Malignant neoplasm of parotid gland |  |
| C08 Malignant neoplasm of other and unspecified major salivary glands |  |
| C09 Malignant neoplasm of tonsil |  |
| C10 Malignant neoplasm of oropharynx |  |
| C11 Malignant neoplasm of nasopharynx |  |
| C12 Malignant neoplasm of piriform sinus |  |
| C13 Malignant neoplasm of hypopharynx |  |
| C14 Malignant neoplasm of other and ill-defined sites in the lip, oral cavity and pharynx |  |
| C15 Malignant neoplasm of oesophagus |  |
| C16 Malignant neoplasm of stomach |  |
| C17 Malignant neoplasm of small intestine |  |
| C18 Malignant neoplasm of colon |  |
| C19 Malignant neoplasm of rectosigmoid junction |  |
| C20 Malignant neoplasm of rectum |  |
| C21 Malignant neoplasm of anus and anal canal |  |
| C22 Malignant neoplasm of liver and intrahepatic bile ducts |  |
| C23 Malignant neoplasm of gallbladder |  |
| C24 Malignant neoplasm of other and unspecified parts of biliary tract |  |
| C25 Malignant neoplasm of pancreas |  |
| C26 Malignant neoplasm of other and ill-defined digestive organs |  |
| C30 Malignant neoplasm of nasal cavity and middle ear |  |
| C31 Malignant neoplasm of accessory sinuses |  |
| C32 Malignant neoplasm of larynx |  |
| C33 Malignant neoplasm of trachea |  |
| C34 Malignant neoplasm of bronchus and lung |  |
| C37 Malignant neoplasm of thymus |  |
| C38 Malignant neoplasm of heart, mediastinum and pleura |  |
| C39 Malignant neoplasm of other and ill-defined sites in the respiratory system and intrathoracic organs |  |
| C40 Malignant neoplasm of bone and articular cartilage of limbs |  |
| C41 Malignant neoplasm of bone and articular cartilage of other and unspecified sites |  |
| C43 Malignant melanoma of skin |  |
| C44 Other malignant neoplasms of skin |  |
| C45 Mesothelioma |  |
| C46 Kaposi's sarcoma |  |
| C47 Malignant neoplasm of peripheral nerves and autonomic nervous system |  |
| C48 Malignant neoplasm of retroperitoneum and peritoneum |  |
| C49 Malignant neoplasm of other connective and soft tissue |  |
| C50 Malignant neoplasm of breast | X |
| C51 Malignant neoplasm of vulva | X |
| C52 Malignant neoplasm of vagina | X |
| C53 Malignant neoplasm of cervix uteri | X |
| C54 Malignant neoplasm of corpus uteri | X |
| C55 Malignant neoplasm of uterus, part unspecified | X |
| C56 Malignant neoplasm of ovary | X |
| C57 Malignant neoplasm of other and unspecified female genital organs | X |
| C58 Malignant neoplasm of placenta | X |
| C60 Malignant neoplasm of penis | X |
| C61 Malignant neoplasm of prostate | X |
| C62 Malignant neoplasm of testis | X |
| C63 Malignant neoplasm of other and unspecified male genital organs | X |
| C64 Malignant neoplasm of kidney, except renal pelvis |  |
| C65 Malignant neoplasm of renal pelvis |  |
| C66 Malignant neoplasm of ureter |  |
| C67 Malignant neoplasm of bladder |  |
| C68 Malignant neoplasm of other and unspecified urinary organs |  |
| C69 Malignant neoplasm of eye and adnexa |  |
| C70 Malignant neoplasm of meninges |  |
| C71 Malignant neoplasm of brain |  |
| C72 Malignant neoplasm of spinal cord, cranial nerves and other parts of central nervous system |  |
| C73 Malignant neoplasm of thyroid gland |  |
| C74 Malignant neoplasm of adrenal gland |  |
| C75 Malignant neoplasm of other endocrine glands and related structures |  |
| C76 Malignant neoplasm of other and ill-defined sites |  |
| C77 Secondary and unspecified malignant neoplasm of lymph nodes |  |
| C78 Secondary malignant neoplasm of respiratory and digestive organs |  |
| C79 Secondary malignant neoplasm of other sites |  |
| C80 Malignant neoplasm without specification of site |  |
| C81 Hodgkin's disease |  |
| C82 Follicular [nodular] non-Hodgkin's lymphoma |  |
| C83 Diffuse non-Hodgkin's lymphoma |  |
| C84 Peripheral and cutaneous T-cell lymphomas |  |
| C85 Other and unspecified types of non-Hodgkin's lymphoma |  |
| C86 Other specified types of T/NK cell lymphoma |  |
| C88 Malignant immunoproliferative diseases |  |
| C90 Multiple myeloma and malignant plasma cell neoplasms |  |
| C91 Lymphoid leukaemia |  |
| C92 Myeloid leukaemia |  |
| C93 Monocytic leukaemia |  |
| C94 Other leukaemias of specified cell type |  |
| C95 Leukaemia of unspecified cell type |  |
| C96 Other and unspecified malignant neoplasms of lymphoid, haematopoietic and related tissue |  |
| C97 Malignant neoplasms of independent (primary) multiple sites |  |
| D00 Carcinoma in situ of oral cavity, oesophagus and stomach |  |
| D01 Carcinoma in situ of other and unspecified digestive organs |  |
| D02 Carcinoma in situ of middle ear and respiratory system |  |
| D03 Melanoma in situ |  |
| D04 Carcinoma in situ of skin |  |
| D05 Carcinoma in situ of breast | X |
| D06 Carcinoma in situ of cervix uteri | X |
| D07 Carcinoma in situ of other and unspecified genital organs | X |
| | D09 Carcinoma in situ of other and unspecified sites | | --- | |  |
| D10 Benign neoplasm of mouth and pharynx |  |
| D11 Benign neoplasm of major salivary glands |  |
| D12 Benign neoplasm of colon, rectum, anus and anal canal |  |
| D13 Benign neoplasm of other and ill-defined parts of digestive system |  |
| D14 Benign neoplasm of middle ear and respiratory system |  |
| D15 Benign neoplasm of other and unspecified intrathoracic organs |  |
| D16 Benign neoplasm of bone and articular cartilage |  |
| D17 Benign lipomatous neoplasm |  |
| D18 Haemangioma and lymphangioma, any site |  |
| D19 Benign neoplasm of mesothelial tissue |  |
| D20 Benign neoplasm of soft tissue of retroperitoneum and peritoneum |  |
| D21 Other benign neoplasms of connective and other soft tissue |  |
| D22 Melanocytic naevi |  |
| D23 Other benign neoplasms of skin |  |
| D24 Benign neoplasm of breast | X |
| D25 Leiomyoma of uterus | X |
| D26 Other benign neoplasms of uterus | X |
| D27 Benign neoplasm of ovary | X |
| D28 Benign neoplasm of other and unspecified female genital organs | X |
| D29 Benign neoplasm of male genital organs | X |
| D30 Benign neoplasm of urinary organs |  |
| D31 Benign neoplasm of eye and adnexa |  |
| D32 Benign neoplasm of meninges |  |
| D33 Benign neoplasm of brain and other parts of central nervous system |  |
| D34 Benign neoplasm of thyroid gland |  |
| D35 Benign neoplasm of other and unspecified endocrine glands |  |
| D36 Benign neoplasm of other and unspecified sites |  |
| D37 Neoplasm of uncertain or unknown behaviour of oral cavity and digestive organs |  |
| D38 Neoplasm of uncertain or unknown behaviour of middle ear and respiratory and intrathoracic organs |  |
| D39 Neoplasm of uncertain or unknown behaviour of female genital organs | X |
| D40 Neoplasm of uncertain or unknown behaviour of male genital organs | X |
| D41 Neoplasm of uncertain or unknown behaviour of urinary organs |  |
| D42 Neoplasm of uncertain or unknown behaviour of meninges |  |
| D43 Neoplasm of uncertain or unknown behaviour of brain and central nervous system |  |
| D44 Neoplasm of uncertain or unknown behaviour of endocrine glands |  |
| D45 Polycythaemia vera |  |
| D46 Myelodysplastic syndromes |  |
| D47 Other neoplasms of uncertain or unknown behaviour of lymphoid, haematopoietic and related tissue |  |
| D48 Neoplasm of uncertain or unknown behaviour of other and unspecified sites |  |
