# Supplementary material for: The age pattern of the male-to-female ratio in mortality from COVID-19 mirrors that of cardiovascular disease in the general population
Source: Aging (Albany NY). 2021 Feb 7;13(3):3190–201. doi: 10.18632/aging.202639 (PMC7906174; doi:10.18632/aging.202639)
Supplement: Supplementary Tables 4, 5 and 6 [file aging-13-202639-s004.pdf]

SUPPLEMENTARY TABLES

Supplementary Table 4. Raw data for cardiovascular disease deaths stratified by age and sex.

Country	Year	Sex	0-9	10-19	20-29	30-39	40-49	50-59	60-69	70-79	80-89	90+
Netherlands	2016	F	1	0	4	15	92	248	600	1523	3953	2715
Netherlands	2016	M	0	1	9	26	150	524	1371	2414	3410	1091
Germany	2015	F	7	11	33	86	488	1654	4004	15749	41202	30116
Germany	2015	M	6	8	52	247	1473	5720	11281	27166	35472	10437
Italy	2015	F	11	6	23	73	278	828	2300	8775	32644	28310
Italy	2015	M	10	11	39	207	954	2698	6127	13858	26324	11478
Spain	2015	F	1	2	10	66	245	583	1288	4087	14197	10084
Spain	2015	M	5	10	30	160	780	2286	4011	7396	12333	4629
France	2014	F	9	7	17	55	298	710	1449	3778	13798	11762
France	2014	M	11	6	31	175	790	2181	4417	6500	12377	5088
**Country**	**Year**	**Sex**	**0-4**	**5-14**	**15-24**	**25-34**	**35-44**	**45-54**	**55-64**	**65-74**	**75-84**	**85+**
United States of America	2015	F	63	45	112	515	2069	7714	18751	32406	58308	119546
United States of America	2015	M	86	51	187	1029	4585	17712	42256	57005	68928	75730

Supplementary Table 5. Raw data for cancer deaths (all) stratified by age and sex.

Country	Year	Sex	30-39	40-49	50-59	60-69	70-79	80-89	90+
Netherlands	2016	M	115	607	2178	5986	8205	6844	1324
Netherlands	2016	F	201	698	2452	4802	5789	5700	1893
Germany	2015	M	592	2960	13167	26374	45208	32306	5315
Germany	2015	F	723	3191	10623	18174	32371	31501	10438
Italy	2015	M	541	2302	7636	19063	32100	31181	6175
Italy	2015	F	613	2707	6776	12629	20792	26554	9083
Spain	2015	M	468	1885	7476	14435	19171	19631	4339
Spain	2015	F	483	1972	4891	6883	9573	14533	5120
France	2014	M	603	2693	11210	23238	23756	25318	6337
France	2014	F	701	2736	7229	12433	14723	22025	9367
**Country**	**Year**	**Sex**	**25-34**	**35-44**	**45-54**	**55-64**	**65-74**	**75-84**	**85+**
United States of America	2015	M	1910	4791	21551	65113	92333	84381	50707
United States of America	2015	F	1945	6379	22185	52637	74555	73740	57043

Supplementary Table 6. Raw data for cancer deaths (excluding sex-biased cancers) stratified by age and sex.

Country	Year	Sex	30-39	40-49	50-59	60-69	70-79	80-89	90+
Netherlands	2016	M	111	597	2106	5575	7294	5686	1042
Netherlands	2016	F	117	415	1684	3651	4567	4560	1455
Germany	2015	M	563	2895	12726	24698	40057	26589	3906
Germany	2015	F	425	1814	7037	13125	23680	23879	7973
Italy	2015	M	520	2254	7475	18327	29942	27450	4929
Italy	2015	F	370	1480	4328	9010	15972	21157	7176
Spain	2015	M	449	1853	7334	13858	17676	16814	3345
Spain	2015	F	278	1094	3326	4914	7335	11435	4036
France	2014	M	576	2659	11009	22242	21735	21342	4744
France	2014	F	400	1563	4864	8524	10504	16682	7284
**Country**	**Year**	**Sex**	**25-34**	**35-44**	**45-54**	**55-64**	**65-74**	**75-84**	**85+**
United States of America	2015	M	1800	4686	21006	62199	85570	74508	40933
United States of America	2015	F	1192	3456	13893	36710	56325	58977	45779
